# Supplementary material for: Phytochemical Composition and Bioactive Potential of Melissa officinalis L., Salvia officinalis L. and Mentha spicata L. Extracts
Source: Foods. 2023 Feb 23;12(5):947. doi: 10.3390/foods12050947 (PMC10000423; doi:10.3390/foods12050947)
Supplement: Supplementary file 1 [file foods-12-00947-s001.zip › foods-2201769-Supplementary.pdf]

Table S1. Limit of detection (LOD), limit of quantification (LOQ) and coefficient of linear correlation (R^2) of the different standards used to obtain the calibration curves required for phenolic compound quantification

Compound Standard	LOD (μg/mL)	LOQ (μg/mL)	R^2
4-Hydroxybenzoic acid	0.17	1.22	0.999
Apigenin-6-*C*-glucoside	0.19	0.63	0.9989
Apigenin-7-*O*-glucoside	0.10	0.53	0.999
Caffeic acid	0.78	1.97	0.994
Chlorogenic acid	0.20	0.68	0.9999
Naringenin	0.20	0.64	0.9998
Rosmarinic acid	0.15	0.68	0.999

Table S2. Phenolic compound content (mg/g dry extract) of sage (*Salvia officinalis* L.) extracts

Peak	Rt	λmax	[M-H] *m/z*	MS²	Tentative Identification	Decoction	Infusion	Hydroethanolic
**1**	4.87	267sh319	311	179(60),149(100),135(9)	Caftaric acid [1]	0.24±0.01	0.27±0.01	0.264±0.001
**2**	5.15	325	503	341(17),281(15),221(8),179(34),161(38),135(5)	6-*O*-Caffeoyl-fructosyl-glucoside [4]	0.28±0.02	0.27±0.02	0.23±0.01
**3**	5.74	328	341	179(100)	Caffeic acid hexoside [4]	0.15±0.01	0.141±0.004	0.115±0.002
**4**	7.30	325	387	369(26), 207(100), 163(47)	Caffeic acid acetylhexoside [4]	0.11±0.01	0.096±0.004	0.119±0.005
**5**	7.77	282sh323	377	207(100),191(5),163(38),137(7)	3-*p*-Coumarouylquinic acid [5]	0.1±0.01	0.1±0.002	0.088±0.002
**6**	8.8	337	593	473(18), 383(6), 353(12)	Apigenin-6,8-di-*C*-hexoside [2]	1.23±0.09	1.42±0.08	0.89±0.02
**7**	10.96	323	537	519(84),341(10),179(32),161(48),135(10)	Salvianolic acid I [7]	0.7±0.02	0.67±0.03	0.55±0.01
**8**	13.14	325	637	285(100)	Luteolin-*O*-di-glucuronide [3]	1.1±0.07	1.33±0.08	1.05±0.05
**9**	14.87	341	477	301(100)	6-Hydroxyluteolin-7-*O*-glucuronide [7]	3.54±0.25	3.63±0.17	3.05±0.03
**10**	15.93	327	597	359(30),295(26),197(16),179(15),135(8)	Yunnaneic acid F [7]	0.93±0.02	0.87±0.07	0.66±0.01
**11**	17.18	341	593	473(21), 383(9), 353(16)	Apigenin 6-*C*-glucose-8-*C*-glucose [7]	1±0.03	1.47±0.01	0.837±0
**12**	17.81	345	461	285(100)	Luteolin-7-*O*-glucuronide [3]	18.07±0.71	15.7±0.98	12.06±0.41
**13**	19.41	337	717	537(78),519(100),493(53),339(27),321(45)295(62)	Salvianolic acid B [7]	1.66±0.04	2.88±0.11	2.08±0.02
**14**	20.66	336	359	359(47),197(73),179(71),161(100)	*cis*-Rosmarinic acid [7]	28.4±1.25	24.72±1.29	22.72±0.71
**15**	21.22	289sh326	555	493(100),359(16),225(5)	Salvianolic acid K isomer I [7]	4.46±0.13	3.64±0.15	3.34±0.09
**16**	22.09	329	555	493(100),359(16),225(5)	Salvianolic acid K isomer II [7]	2.98±0.12	3.34±0.24	2.31±0.08
**17**	22.64	334	447	285(100)	Luteolin-*O*-hexoside isomer I [3]	6.2±0.1	5.33±0.34	3.48±0.1
**18**	23.36	332	359	359(45),197(69),179(75),161(100)	*trans*-Rosmarinic acid [7]	1.5±0.08	2.05±0.12	1.39±0.05
**19**	24.26	330	555	493(100),359(26),225(7)	Salvianolic acid K isomer [7]	1.71±0.02	1.66±0.02	1.36±0.05

Table S2 *(cont.)*. Phenolic compound content (mg/g dry extract) of sage (*Salvia officinalis* L.) extracts

Peak	Rt	λmax	[M-H] *m/z*	MS²	Tentative Identification	Decoction	Infusion	Hydroethanolic
**20**	25.77	336	503	285(100)	Luteolin-acetyl-*O*-glucuronide [3]	3.52±0.08	3.07±0.07	2.55±0.2
**21**	27.22	328	537	493(100),359(43),313(8),295(5)	Lithospermic acid A [7]	3.32±0.12	2.69±0.11	1.99±0.02
**22**	27.9	327	563	545(27),503(40),473(100),443(83),383(90),353(85)	Apigenin 6-*C*-pentosyl-8-*C*-hexoside [2]	1.14±0.01	0.881±0.001	0.73±0.04
**23**	30.86	337	447	285(100)	Luteolin-*O*-hexoside isomer II [3]	1.16±0.02	0.9±0.01	0.87±0.05
**24**	32.87	283sh323	717	537(25),519(44),493(18),339(24),321(25),313(9),295(100)	Salvianolic acid E [7]	0.58±0.04	0.54±0.01	0.44±0.01
					**Total Phenolic Acids**	47.12±1.38	43.93±0.61	37.64±0.77
					**Total Flavonoids**	36.95±1.18	33.74±1.71	25.53±0.24
					**Total Phenolic Compounds**	84.07±2.56	77.67±1.1	63.17±1.01

Rt: Retention time (min), λ_{max}: wavelengths of maximum absorption in the visible region (nm); MS²: second stage of mass spectrometry. Superscript numbers indicate the compound standard used for the quantification: (1) 4-Hydroxybenzoic acid; (2) Apigenin-6-*C*-glucoside; (3) Apigenin-7-*O*-glucoside; (4) Caffeic acid; (5) Chlorogenic acid; (6) Naringenin; (7) Rosmarinic acid. Content values expressed as mean ± SD (n= 3).

Table S3. Phenolic compound content (mg/g dry extract) of lemon balm (*Melissa officinalis* L.) extracts

Peak	Rt	λ_{max}	[M-H] *m/z*	MS^2	Tentative Identification	Decoction	Infusion	Hydroethanolic
**1**	4.67	328	311	179(61),149(100),135(8)	Caftaric acid [1]	0.22±0.01	0.39±0.01	nd
**2**	8.86	288sh324	179	135(100)	Caffeic acid [4]	0.102±0.004	0.037±0.002	0.366±0.005
**3**	9.27	281sh325	539	495(63),359(21),295(45),279(4),197(34),179(16)	Yunnaneic acid D [7]	0.32±0.01	0.31±0.01	0.15±0.01
**4**	11.56	323	537	519(84),341(10),179(32),161(48),135(10)	Salvianolic acid I [7]	1.31±0.07	1.18±0.02	0.332±0.001
**5**	15.53	284sh326	623	461(100), 285(85)	Luteolin-*O*-hexosyl-*O*-glucuronide [3]	0.62±0.04	0.59±0.01	0.611±0.003
**6**	16.35	330	521	359(51), 197(21),179(37),161(100)	Rosmarinic acid hexoside [7]	2.36±0.01	1.83±0.08	1.36±0.01
**7**	16.9	278sh319	717	519(100), 339, 321	Salvianolic acid A [7]	1.27±0.05	1.78±0.06	0.53±0.02
**8**	18.59	283sh325	719	539(12),521(9), 359(100), 197(9), 179(12), 161(51),135(5)	Sagerinic acid [7]	2.35±0.15	2.03±0.06	1.082±0.005
**9**	19.91	330	439	359(10), 179(8),161(40),135(28)	Sulphated rosmarinic acid [7]	1.37±0.07	0.95±0.07	0.67±0.03
**10**	20.57	316sh331	359	197(81),179(90),161(100), 135(28)	Rosmarinic acid [7]	41.71±2	34.4±0.2	40.36±0.22
**11**	24.3	326	537	493(100),359(35),313(4),295(2)	Lithospermic acid A [7]	11.51±0.81	5.78±0.36	4.54±0.04
**12**	27.17	328	537	493(100),359(32),313(4),295(5)	Lithospermic acid A isomer [7]	16.09±0.06	9.44±0.51	6.04±0.41
**13**	30.05	328	829	667(82), 535(100), 491(21),311(50), 293(5), 197(3), 179(11)	Salvianolic acid C derivative [7]	3.76±0.1	1.51±0.04	1.43±0.03
**14**	33.16	287sh324	717	537(25),519(44),493(18),339(24),321(25),313(9),295(100)	Salvianolic acid E [7]	1.53±0.04	0.75±0.02	0.86±0.01
					**Total Phenolic Acids**	83.9±1.74	60.41±1.41	57.74±0.31
					**Total Flavonoids**	0.62±0.04	0.59±0.01	0.611±0.003
					**Total Phenolic Compounds**	84.51±1.77	61±1.42	58.35±0.31

Rt: Retention time (min), λ_{max}: wavelengths of maximum absorption in the visible region (nm); MS^2: second stage of mass spectrometry. Superscript numbers indicate the compound standard used for the quantification: (1) 4-Hydroxybenzoic acid; (2) Apigenin-6-*C*-glucoside; (3) Apigenin-7-*O*-glucoside; (4) Caffeic acid; (5) Chlorogenic acid; (6) Naringenin; (7) Rosmarinic acid. Content values expressed as mean ± SD (n= 3); nd: not detected.

Table S4. Phenolic compound content (mg/g dry extract) of spearmint (*Mentha spicata* L.) extracts

Peak	Rt	Λmax	[M-H] *m/z*	MS²	Tentative Identification	Decoction	Infusion	Hydroethanolic
**1**	3.95	281	191	173(100),111(23)	Quinic acid [1]	0.61±0.01	0.222±0.003	nd
**2**	4.67	283sh321	387	193(100),179(3),161(8),133(4)	Caffeic acid methyl ester dimer [4]	nd	0.01±0.01	0.101±0.002
**3**	5.91	291sh314	353	191(20), 179(61), 173(100). 135(12)	5-*O*-Caffeoylquinic acid [5]	0.86±0.03	0.03±0.03	0.047±0.002
**4**	8.96	299sh321	179	135(100)	Caffeic acid [4]	0.25±0.002	1.01±0.03	1.23±0.07
**5**	12.67	284sh326	623	461(100), 285(84)	Luteolin-*O*-hexosyl-*O*-glucuronide [3]	0.57±0.01	0.07±0.002	0.07±0.002
**6**	13.89	285sh331	595	287(100)	Eriodictyol-7-*O*-rutinoside [5]	1.84±0.03	3.99±0.11	4.25±0.09
**7**	16.87	347	593	285(100)	Luteolin-*O*-rutinoside [3]	9.29±0.06	1.26±0.03	2.34±0.11
**8**	17.91	347	461	285(100)	Luteolin-*O*-glucuronide [3]	15.29±0.05	3.56±0.09	4.91±0.18
**9**	19.55	285sh338	717	519(100), 339, 321	Salvianolic acid A [7]	2.197±0.005	1.22±0.04	1.19±0.03
**10**	20.62	331	359	197(87), 179(91), 161(100), 135(48)	Rosmarinic acid [7]	31.27±0.35	19.61±0.49	32.08±1.18
**11**	22.58	282sh326	719	539(12),521(9), 359(100), 197(9), 179(12), 161(51),135(5)	Sagerinic acid [7]	3.29±0.14	2.24±0.08	2.68±0.01
**12**	24.34	299sh327	537	493(100),359(35),313(4),295(2)	Lithospermic acid A [7]	5.89±0.41	2.83±1.3	5.57±0.02
**13**	27.08	289sh324	533	489 (100), 285 (51)	Luteolin-7-*O*-malonylglucoside [3]	2.19±0.11	0.39±0.08	0.58±0.04
**14**	28.96	298sh325	537	493(70), 359(15), 313(30), 295(100),269(25),197(16),179(72)	Lithospermic acid A isomer [7]	3.64±0.22	2.35±0.01	2.87±0.05
					**Total Phenolic Acids**	48.01±0.41	29.52±0.78	45.77±1.24
					**Total Flavonoids**	29.19±0.1	9.28±0.16	12.15±0.34
					**Total Phenolic Compounds**	77.2±0.51	38.79±0.62	57.92±1.59

Rt: Retention time (min), λ_{max}: wavelengths of maximum absorption in the visible region (nm); MS²: second stage of mass spectrometry. Superscript numbers indicate the compound standard used for the quantification: (1) 4-Hydroxybenzoic acid; (2) Apigenin-6-*C*-glucoside; (3) Apigenin-7-*O*-glucoside; (4) Caffeic acid; (5) Chlorogenic acid; (6) Naringenin; (7) Rosmarinic acid. Content values expressed as mean ± SD (n= 3); nd: not detecte
